# Delirium Following the Use of Tartar Emetic

**Published:** 1844-11-16

**Authors:** 


DELIRIUM FOLLOWING THE USE OF TARTAR EMETIC.

Mr. Whittaker was in attendance on a gentleman affected with pneumonia, which he treated with bleeding, and the exhibition of small doses of tartarized antimony. By these means the disease was subdued, but a new train of symptoms set in; the patient became in a half frantic state, with a countenance of health, yet expressive of much pleasurable excitement; tremors of the hands, picking of the bed-clothes, and every thing that came within reach; unusual activity, great volubility of speech, and extraordinary craftiness. This state, which Mr. Whittaker designates delirium tremens, but which appears to us more to resemble mania, was successfully treated by the exhibition of solid opium in large doses.—*Ibid.*

